# Enhancing Biomass and Lutein Production From *Scenedesmus almeriensis*: Effect of Carbon Dioxide Concentration and Culture Medium Reuse

**DOI:** 10.3389/fpls.2020.00415

**Published:** 2020-04-21

**Authors:** Antonio Molino, Sanjeet Mehariya, Angela Iovine, Patrizia Casella, Tiziana Marino, Despina Karatza, Simeone Chianese, Dino Musmarra

**Affiliations:** ^1^Department of Sustainability-CR Portici, ENEA Italian National Agency for New Technologies, Energy and Sustainable Economic Development, Portici, Italy; ^2^Department of Engineering, University of Campania “Luigi Vanvitelli”, Aversa, Italy

**Keywords:** microalgae, photo-autotrophic cultivation, *Scenedesmus almeriensis*, lutein, ASE

## Abstract

The main purpose of this study is to investigate the effects of operative parameters and bioprocess strategies on the photo-autotrophic cultivation of the microalgae *Scenedesmus almeriensis* for lutein production. *S. almeriensis* was cultivated in a vertical bubble column photobioreactor (VBC-PBR) in batch mode and the bioactive compounds were extracted by accelerated solvent extraction with ethanol at 67°C and 10 MPa. The cultivation with a volume fraction of CO_2_ in the range 0–3.0%v/v showed that the highest biomass and lutein concentrations – 3.7 g/L and 5.71 mg/g, respectively – were measured at the highest CO_2_ concentration and using fresh growth medium. Recycling the cultivation medium from harvested microalgae resulted in decreased biomass and lutein content. The nutrient chemical composition analysis showed the highest consumption rates for nitrogen and phosphorus, with values higher than 80%, while sulfate and chloride were less consumed.

## Introduction

Lutein is a carotenoid found in plants and phototrophic microorganisms. It is classified as a primary xanthophyll because of the presence of two hydroxyl functional groups in its chemical structure (Chen et al., 2017; Xie et al., 2017; Bowen et al., 2018). Lutein acts as a light-harvesting pigment that improves photosynthesis efficiency and prevents photo-damage in plant cells (Ruban et al., 2007). For its outstanding antioxidant, anti-inflammatory and colorant properties, lutein is widely used as a nutraceutical for human health (Fernández-Sevilla et al., 2010). Lutein is used in the treatment of age-related macular degeneration, and for the prevention of cardiovascular diseases and some types of cancers (Coleman and Chew, 2007; Cerón et al., 2008). The existing commercial source of natural lutein is marigold (*Tagetes erecta L.*) petals (Lin et al., 2015b). However, the lutein content of marigold petals is around 0.03% (based on dry biomass weight), and their harvesting and separation carry a high cost (Lin et al., 2015a). Therefore, there is a need for an alternative source for cost-effective production.

Microalgae have been considered as a potential source of natural lutein as their pigment content (0.5-1.2% based on dry biomass weight) is far higher than that of conventional sources (Cerón et al., 2008; Sánchez et al., 2008b; Lin et al., 2015a, b; Chen et al., 2017). Additionally, producing lutein from microalgae offers several environmental benefits, such as a carbon dioxide mitigation as their photosynthesis efficiency is 10-15 times higher than that of terrestrial plants (Lam et al., 2012; Yen et al., 2013; Xie et al., 2014). The microalgae growth rate is 5–10 times higher than that of higher plants. They can be grown in seawater and brackish water, as well as on non-arable land, therefore they do not compete with conventional agriculture crops for resources (Del Campo et al., 2001; Utomo et al., 2013; Ho et al., 2014; Lin et al., 2015a). *Scenedesmus almeriensis* is recognized as a rich source of lutein, containing up to 4.5 mg/g (dry weight), when grown in outdoor culture conditions (Del Campo et al., 2007); moreover, lutein content can be increased by manipulating growth conditions, such as light intensity and temperature, till to 5.4 mg/g (dry weight) (Sánchez et al., 2008b).

Christian et al. (2018) repot that the astaxanthin content of *Haematococcus pluvialis* using high concentrations of CO_2_ (15%v/v) as the carbon source can achieve the value of about 36 mg/g (dry weight). Cheng et al. (2016) observed the highest biomass (0.65 g/L) and astaxanthin (45 mg/L) concentrations in *Haematococcus pluvialis* grown in 6% CO_2_.

Microalgae cultivation requires large amounts of water and nutrients, which reduces the cost-effectiveness of the entire bio-compound extraction process (Hadj-Romdhane et al., 2013). The re-use of culture media could be a solution for the development of large-scale cultures to minimize water use and nutrient consumption (Fret et al., 2017). For the cultivation of *Scenedesmus obliquus*, Livansky et al. (1996) found that water use and nutrient consumption could be reduced of about 64 and 16%, respectively, by recycling the growth medium. For *Chlorella vulgaris* Hadj-Romdhane et al. (2012) reported that the use of optimized culture conditions during medium recycling could decrease water use and nutrient consumption of 75 and 62%, respectively.

As CO_2_ is used as the carbon source, microalgae cultivation also contributes to CO_2_ sequestration, which makes it one of the most promising approaches to deal with global warming (Salih, 2011; Chen and Liu, 2018). Not only does the higher CO_2_ concentration increase the carbon source available for the growth of microalgae, but it also improves the assimilation of nutrients in their biomass (de Assis et al., 2019). The microalgae can efficiently convert atmospheric CO_2_ into organic biomass via carbon fixation. Several inexpensive sources of CO_2_ can be explored, such as CO_2_ -rich industrial exhaust gases and fermentation effluent gases that could make the microalgae cultivation process cost-effective and eco-friendly (Lam et al., 2012; Xie et al., 2014; Chen and Liu, 2018). Patel et al. (2019) cultivated *Chlorella protothecoides* in 5% of CO_2_ and obtained 4.12 g/L of biomass. Xie et al. (2017) cultivated *Desmodesmus* sp. F51 and evaluated the effect of CO_2_ concentration on microalgae biomass and lutein production. Six different CO_2_ concentrations (0.03, 2.5, 5.0, 7.5, 10.0, and 12.5%) were used. The biomass productivity and the specific growth rate were higher when CO_2_ concentration increased from 0.03 to 2.5% and decreased when CO_2_ concentration was further increased to 12.5% (Xie et al., 2017). On the basis of the above-mentioned studies 3.0% was chosen as maximum value to evaluate the effect of CO_2_ concentration in this study.

Moheimani (2016) showed that *Tetraselmis suecica* can be grown in CO_2_ from a coal-fired power plant flue gas and by reusing the growth medium. CO_2_ biofixation with nutrient recycling and the addition of monoethanolamine were tested on *Spirulina* sp. cultivation by da Rosa et al. (2015). They highlighted that *Spirulina* can be produced using recycled medium, in spite of a reduce protein and lipid content. Cui et al. (2019) showed that using flue gas from a biomass plant and recycling the growth to cultivate *Spirulina* sp. a 42% lower nutrient consumption was achieved, with no significant differences between fresh medium and recycled medium in terms of protein and phycocyanin contents.

To the best of the authors knowledge, the combined effect of CO_2_ concentration and medium recycling on the *S. almeriensis* growth and lutein production has not been investigated anywhere before. The present study aims at this dual purpose: developing a lab-scale methodology to recycle the supernatant/filtrate growth medium obtained from the harvesting of the microalgae biomass, and assessing how different CO_2_ concentrations (0–3%v/v) and fresh and recycled growth media influence biomass and lutein production. The biomass was harvested by filtration, then lutein was obtained by accelerated solvent extraction (ASE) at 67°C and 10 MPa. Ethanol, a green solvent belonging to the class of the Generally Recognized as Safe (GRAS) solvents, was used during the extraction step. The lutein content was measured by u-HPLC.

## Materials and Methods

### Microalgae and Growth Medium

Seed culture of *S. almeriensis* was provided by AlgaRes Srl (Rome, Italy), and used for the cultivation under laboratory conditions. Microalgae cells were cultivated in a modified Mann & Myers medium (Mann and Myers, 1968; Barceló-Villalobos et al., 2019), consisting of NaNO_3_ (1.0 g/L), K_2_HPO_4_ (0.1 g/L), MgSO_4_^∗^7H_2_O (1.2 g/L), and CaCl_2_ (0.3 g/L).

Moreover, 10 mL of a solution of micronutrients, containing Na_2_EDTA (0.001 mg/L), MnCl_2_ (1.4 mg/L), ZnSO_4_^∗^7H_2_O (0.33 mg/L), FeSO_4_^∗^2H_2_O (2 mg/L), CuSO_4_^∗^5H_2_O (0.002 mg/L), and Co(NO_3_)_2_^∗^6H_2_O (0.007 mg/L), were added to 990 mL of the growth medium.

### Photo-Bioreactor

*Scenedesmus almeriensis* was cultivated in a vertical bubble column photo-bioreactor (VBC-PBR), made of plexiglass, with a working volume of 1.25 L (effective height = 680 mm; external diameter = 60 mm; thickness = 10 mm) and with a volume to surface ratio (V/S) of 11.5 L/m^2^. The VBC-PBR was fed with a gaseous mixture (N_2_/O_2_/CO_2_) from tanks and was equipped to a monitor and control system which allowed fine tuning of the gaseous mixture flow rate, the temperature, the pH and the light intensity. The bottom of the reactor was equipped with three sintered steel spargers, installed through 3 fileted holes (1/8′′), to feed the gaseous mixture into the reactor. The gaseous mixture flow rate was regulated by Bronkhorst gas flow controllers^TM^, with a flow control accuracy of 0.5%. The top of the reactor was equipped with a temperature sensor (thermocouple) and with a pH sensor. The temperature control system consisted of an AISI 316L coaxial pipe (diameter = 60.3 mm; thickness = 1 mm) in which water was used as cooling fluid. Thanks to a heat pump, the temperature control system allowed to regulate the temperature inside the reactor with a precision of ±1°C in the range 15–35°C.

**FIGURE 1:**
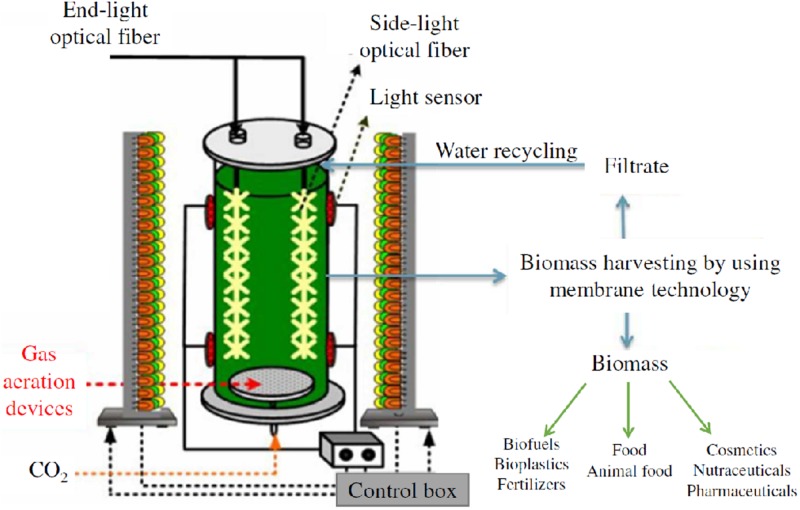
Experimental set-up schematization.

The lighting system consisted of a semi-cylinder structure, located at a distance of 100 mm from the VBC-PBR, with blue, white and red lights from a selective LED system (only blue/only white/only red or a mix of them). The lighting system was controlled and regulated by a SCADA (Supervisory Control and Data Acquisition) system, equipped with a touch-screen, a custom software and a PC to collect and to record experimental data of temperature, gas flow rate, pH, and light intensity. A schematization of the experimental set-up is sketched in Figure 1.

### Growth Conditions

The microalgae were grown under white light with a lux intensity of 4000 lux on the surface of the VBC-PBR, and with a gaseous mixture flow rate (N_2_/O_2_/CO_2_) of 50 mL/min, in which the CO_2_ content was varied in the range 0–3.0%v/v (O_2_ = 21%v/v). The temperature was kept constant at 28°C. The pH of the culture medium changed in the range 7.5–8.5 due to the addition of CO_2_. Two cultivation conditions were investigated. The microalgae were first cultured in the fresh medium from Mann and Myers with the composition described herein (see section “Microalgae and growth medium”). Then around 300 mL of the culture medium were stored to be used as an inoculum in further experiments and the remaining culture was filtered to measure the biomass and lutein content. In the second step, the growth medium recovered by filtration was mixed with the fresh medium and with a given amount of inoculum to achieve an optical density of 0.6–0.7 at 420 nm (see section “Microalgae and growth medium”). In both cases, CO_2_ varied in the range 0–3.0%v/v. Each experimental step was carried out with a working volume of 1.2 L and the compositions of the medium reused are reported in Table 1. Each experimental condition for microalgae growth (expressed as chlorophyll content and biomass concentration), including, nutrient chemical analysis, extraction yield and lutein content measurements, was investigated in replicates, and for each condition, the standard deviation (SD) value was calculated.

**TABLE 1:** Scheme of the inoculum and the medium reuse during the cultivation of *Scenedesmus almeriensis* cultures under batch mode at different cultivation conditions.

				**Growth phase - composed by**		
				
**Experiment**	**Experimental**	**CO_2_ concentration**	**Source of**	**Inoculum**	**Fresh medium**	**Reuse medium**
	**setup**	**(%v/v)**	**inoculum**	**volume**	**volume**	**volume**
A	Fresh medium	Air	Algares	200	1000	0
B	Fresh medium	0.5	Culture transfer after 10 days of growth with air*	1200	0	0
C	Reuse medium	0.5	Culture from 0.5%v/v CO_2_ with fresh medium**	270	550	390
D	Fresh medium	1.5	Culture from 3.0% v/v CO_2_ with reuse medium***	40	1160	0
E	Reuse medium	1.5	Culture from 1.5% v/v CO_2_ with fresh medium****	280	550	370
F	Fresh medium	3	Culture from 0.5% v/v CO_2_ with reuse medium*****	70	1130	0
G	Reuse medium	3	Culture from 3.0% v/v CO_2_ with fresh medium******	230	660	310

*Inoculum derived from sample A; **inoculum derived from sample B; ***inoculum derived from sample G; ****inoculum derived from sample D; *****inoculum derived from sample C; ******inoculum derived from sample F.

### Microalgae Growth Assessment

The *S. almeriensis* cell growth was monitored by determining the absorbance of the samples at 420 nm (Chlorophyll-a), 480 nm (Chlorophyll-b), 690 nm (Chlorophyll-a), and 620 nm (Chlorophyll-b) with a UV/Visible spectrophotometer (Multiskan, Thermo Fisher Scientific, United States), as reported in the literature (Lu et al., 2017; Almomani and Örmeci, 2018; Chirivella-Martorell et al., 2018). The biomass dry weight (BDW) was calculated using the absorbance values at different biomass concentrations measured during the growth phase in the recycled medium. The following calibration curve between absorbance and concentration was obtained:

D⁢B⁢C=(0.0867*A)-0.1868

where DBC is the concentration of biomass on dry weight (g/L) and A is the total absorbance obtained from the sum of the absorbance values at the four chlorophyll wavelengths (Supplementary Figure 1).

For the final dry weight determination, cell cultures were dewatered by vacuum filtration using a vacuum filter with a pore size of 0.45 μm (Sigma-Aldrich, United States) and the pellets were lyophilized for 24 h. Three biological replications were carried out.

### Accelerated Solvent Extraction

Lutein was extracted from mechanically pre-treated *S. almeriensis* cells by the accelerated solvent extraction method, using the Dionex-ASE 200 extractor (Salt Lake City, UT, United States). The pre-treatment was performed via ball milling according to the procedure described elsewhere (Mehariya et al., 2019). Four consecutive extraction cycles were performed using ethanol, a green solvent belonging to the class of the Generally Recognized as Safe (GRAS) solvents, at 67°C and 10 MPa for the complete biomass discoloring. The extraction conditions were optimized elsewhere (Molino et al., 2018c). At the end of each extraction run (20 min), the extracts were collected in 40 mL amber glass vials, by flushing the system with 6.6 mL of fresh solvent, and the system was purged for 1 min with nitrogen (Purity ≥ 99.999%). Five technical replications were carried out.

### Growth Medium Characterization

The chemical analysis of the nutrient concentrations (initial and final) was carried out using an ion Chromatograph (Dionex ICS-1100, Thermo Fisher Scientific, Massachusetts, United States). The Dionex ICS-1100 is an integrated ion chromatography system equipped with a pump, an injection valve, and a conductivity detector. Several nutrients, such as Mg^2^^+^, SO_4_^2^^–^, Na^+^, NO_3_^–^, NO_2_^–^, Ca_2_^+^, Cl^–^, K^+^, and PO_4_^3^^–^ were analyzed.

The extract obtained after each extraction cycle was divided in equal parts and placed in two different vials adding BHT at 0.1wt% as an antioxidant for saponification and gravimetric analysis.

### Lutein Measurement

The total lutein content was gravimetrically quantified after the complete removal of the solvent using a Zymark TurboVap evaporator (Zymark, Hopkinton, MA, United States). Before measuring lutein, the saponification of the samples was carried out in order to remove lipids and chlorophyll, for avoiding the overlap of the spectra with the species present in the carotenoid family (Vechpanich and Shotipruk, 2011; Molino et al., 2018a, b; Sanzo et al., 2018). In particular, the saponification was carried out adding 1 mL of a NaOH solution in methanol (0.05 M) to 5 mL of the extract. This solution was left in the dark in an inert atmosphere for 7 h. Once this step was completed, the sample was neutralized using 3 mL of a NH_4_Cl solution in methanol (0.05 M). After saponification, lutein was measured using a u-HPLC Agilent 1290 Infinity II with Zorbax reverse phase C18 column with the methanol-water (95:5, v/v) mixture as the mobile phase solvent. Flow rate and column temperature were kept constant at 0.4 mL/min and 28°C, respectively. Five technical replications were carried out.

### Statistical Analysis

ANOVA analysis (one-way; α = 0.05) was carried out to compare the results of the effect of the growth medium (fresh and recycled) at different CO_2_ on the biomass productivity, on the nutrient consumption, and on the extraction yield and lutein content.

## Results and Discussion

### CO_2_ Content and Recycled Medium Effects on Chlorophyll Content

**FIGURE 2:**
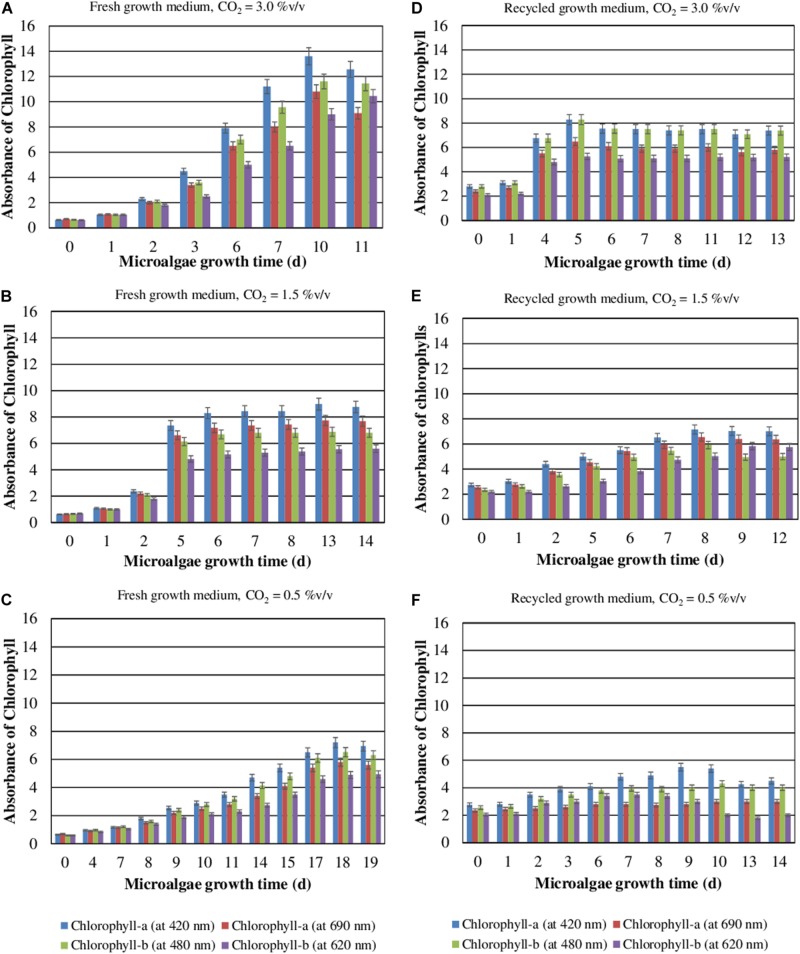
Contents of chlorophyll-a and chlorophyll-b as a function of the *S. almeriensis* growth time at different CO_2_ contents with fresh **(A–C)** and recycled growth medium **(D–F)**. Standard deviation was calculated on three biological replications.

During their autotrophic growth, the microalgae perform photosynthesis using CO_2_ as the inorganic source of carbon. The effects of CO_2_ concentration and medium reuse on the chlorophyll content during the autotrophic cultivation of *S. almeriensis* were evaluated (Figure 2). The results show that the accumulation of chlorophyll increases when CO_2_ content raises and decreases when the medium is recycled. The results also demonstrate that the higher the CO_2_ concentration the higher the chlorophyll content and the lower the time required to reach the peak. The maximum concentrations of chlorophyll-a at 420 nm and chlorophyll-b at 480 nm, 13.6 and 11.6 respectively, were achieved in 10 days with a CO_2_ content of 3.0%v/v and the fresh medium. In addition, the recycled growth medium exhibited a lower photosynthetic efficiency, in terms of chlorophyll content reduction (Figure 2), which could be due to a lower amount of nutrients available. The amount of chlorophyll needed for an efficient light absorption in autotrophic cultivation, where light is the only source of energy, may explain these results. Moreover, the toxic compounds accumulating in the medium during the first culture step may inhibit the photosynthesis efficiency, which may lead to a decrease in the chlorophyll content.

### CO_2_ Content and Recycled Medium Effects on Biomass Concentration

**FIGURE 3:**
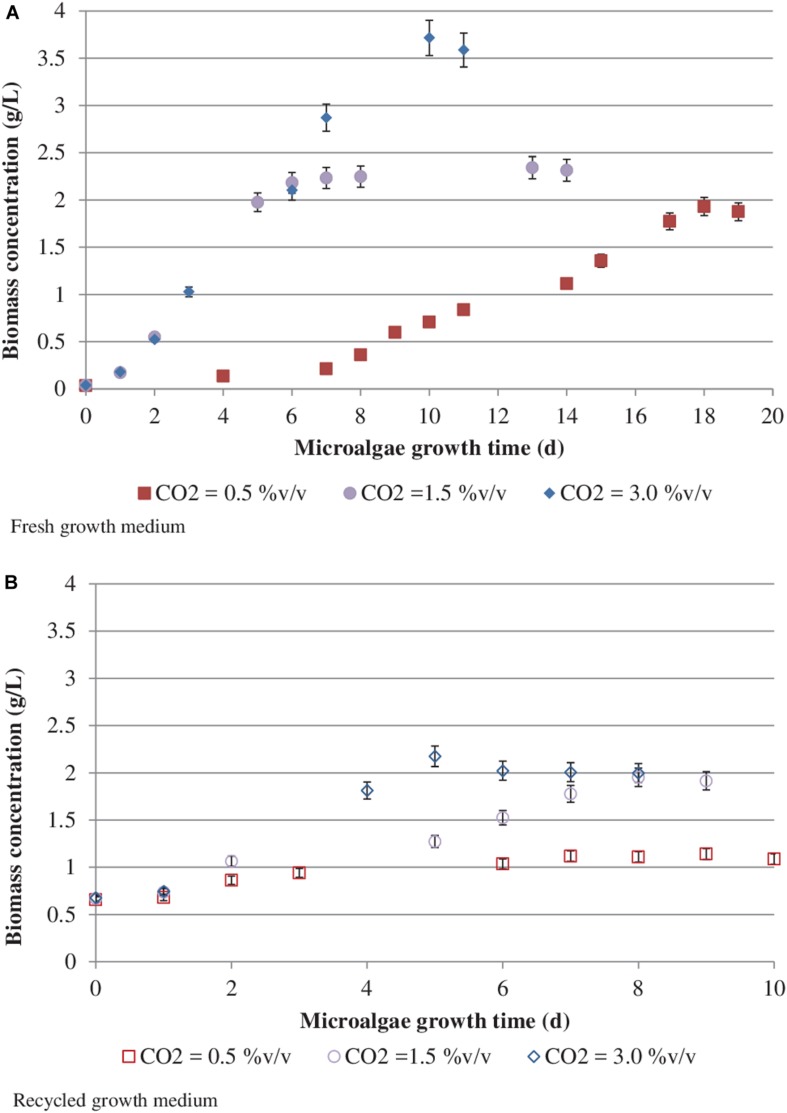
*Scenedesmus almeriensis* concentration (based on dry biomass weight) as a function of growth time at different CO_2_ contents with fresh **(A)** and recycled **(B)** growth medium. Standard deviation was calculated on three biological replications.

**FIGURE 4:**
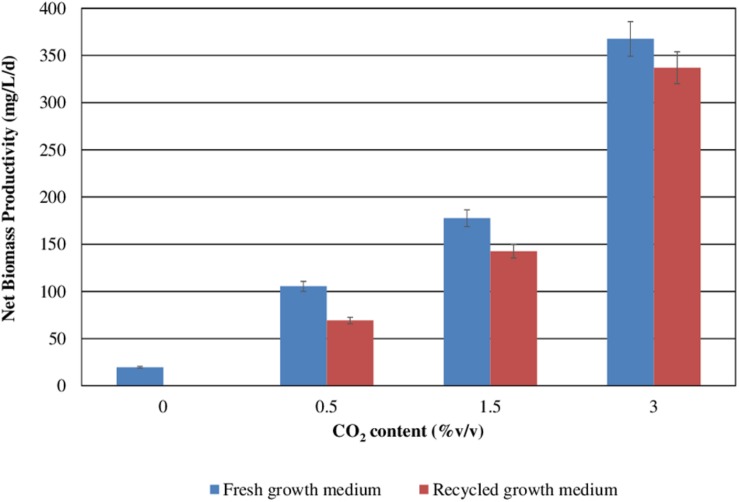
*Scenedesmus almeriensis* productivity (based on dry biomass weight) at different CO_2_ contents with fresh and with recycled growth medium. Standard deviation was calculated on three biological replications.

Figure 3 reports *S. almeriensis* concentration (based on dry biomass weight) as a function of growth time for different CO_2_ contents with fresh and recycled growth medium. Figure 4 reports *S. almeriensis* productivity (based on dry biomass weight) for different CO_2_ contents with fresh and with recycled growth medium. Results in Figure 3 show that with the fresh growth medium, the higher the CO_2_ content the higher the microalgae concentration and the lower the cultivation time. The highest biomass concentration was equal to 3.7 g/L and was achieved by aeration with 3.0%v/v of CO_2_ in 10 days. With the CO_2_ contents of 1.5%v/v and of 0.5%v/v, the biomass concentration was about 2.3 g/L (cultivation time = 14 days) and about 1.9 g/L (cultivation time 18 days), respectively. The biomass concentration decreased when the recycled growth medium was used. After 6 days of cultivation, the reused medium caused saturation in cell growth for all the investigated CO_2_ concentrations. Results in Figure 4 show that *S. almeriensis* productivity markedly increases as CO_2_ concentrations is increased, and evidence that the productivity is higher by using fresh medium than recycled medium. These results agree with those found for continuous cultures of *C. vulgaris*, grown in a recycled medium (Hadj-Romdhane et al., 2012). The recovered medium might lead to an osmotic stress with adverse effects on biomass production and quality (Hadj-Romdhane et al., 2012). Rodolfi et al. (2003) reported that the cultivation of *Nannochloropsis* sp. in fed-batch mode with the recycled medium came to a halt when cell concentration reached about 3 g/L (growth time of 16 days). When the fresh medium was used, the biomass concentration increased above 5 g/L (growth time of 28 days). According to Rodolfi et al. (2003), *Nannochloropsis* sp. released soluble inhibitors and particulate organic matter in the supernatant that seemed to inhibit the cell growth, which could justify the finding of this study. Nevertheless, a statistical analysis (one-way ANOVA with α = 0.05) on the outcomes shown in Figure 4 was performed and the results are reported in Table 2. Significant differences in biomass productivity were observed between fresh and recycled growth media at 0.5% and 1.5% v/v CO_2_ contents. No statistically significant differences were observed at 3% v/v CO_2_ content.

**TABLE 2:** *Scenedesmus almeriensis* productivity (based on dry biomass weight) at different CO_2_ contents with fresh and with recycled growth medium: ANOVA (one-way; α = 0.05) results.

**CO_2_ concentration (%v/v)**	***p*-value**
0.5	0.00049
1.5	0.0017
3	0.088

Both in the presence of the fresh medium and of the recycled medium, the biomass productivity increases concomitantly with the CO_2_ content, passing from about 100 to about 360 mg/L/day with the fresh medium, and from about 75 mg/L/day to about 340 mg/L/day with the recycled medium.

Dineshkumar et al. (2015a) reported that, by increasing CO_2_ concentration from 0.8 to 2.5%, the photosynthesis efficiency improved, which may improve the biomass productivity of *Chlorella minutissima* MCC-27, while a further increase in CO_2_ (>5%) negatively influenced the productivity of microalgae. The optimal value of CO_2_ content, able to enhance the growth rate and the product accumulation in *C. minutissima* MCC- 27, of 3.5%v/v was found by the particle swarm optimization technique. However, it should be noted that the optimal CO_2_ content for microalgae growth usually differs from species to species and depends on the culture medium and the growth conditions. It was found that *Chlorella* sp. and *Nannochloropsis oculata* exhibited an optimal growth with a CO_2_ concentration of 2%v/v and the growth of microalgae was completely inhibited when the CO_2_ was higher than 5%v/v (Chiu et al., 2008, 2009). Liu et al. (2019) found that the dynamics of biomass concentration, and the biomass productivity, was higher in *Arthrospira platensis* cultures aerated with a CO_2_ content of 0.5%, while the growth decreased at lower or higher CO_2_ contents than 0.5%v/v. When CO_2_ contents were higher than 10%v/v, the algal cells only exhibited a slight increase at initial hours to days and finally bleached by the end of experiments. In summary, the optimum CO_2_ level should be identified for each microalgae species to attain the highest biomass productivity (Santos et al., 2018).

### CO_2_ Content and Recycled Medium Effects on Nutrient Consumption

Microalgae growth requires an adequate amount of several nutrients. Among them, nitrogen (N) and phosphorus (P) are essential for the development of cells and their metabolic activity. They are usually used as buffer agents (Choi et al., 2017). In this study, initial and final concentrations of nutrients were measured (Table 3). The chemical analysis shows that the highest nutrient concentrations were found in the fresh medium. The initial concentration of nutrients was slightly different due to the varied nutrient concentration in the inoculum and filtrate.

**TABLE 3:** Initial and final nutrient concentration during the cultivation of *Scenedesmus almeriensis* cultures under batch mode at different cultivation conditions.

	**Concentration (mg/L)**											
	
	**0.5%v/v CO_2_**				**1.5%v/v CO_2_**				**3.0%v/v CO_2_**			
			
	**Fresh medium**		**Reused medium**		**Fresh medium**		**Reused medium**		**Fresh medium**		**Reused medium**	
						
**Nutrients**	**IC**	**FC**	**IC**	**FC**	**IC**	**FC**	**IC**	**FC**	**IC**	**FC**	**IC**	**FC**
Mg_2_^+^	90.25	56.26	80.21	68.88	115.22	58.88	88.41	72.10	111.06	50.55	90.56	70.78
SO_4_^2^^–^	435.21	355.76	440.12	400.56	406.28	378.61	410.58	398.25	448.87	400.25	425.21	411.72
Na^+^	245.23	158.25	167.85	147.25	291.80	150.25	236.85	180.63	270.73	120.35	217.23	160.10
NO_3_^–^	520.38	9.85	333.97	10.52	722.34	10.70	356.25	70.58	630.89	0.00	426.60	11.77
Ca_2_^+^	81.46	57.89	85.07	65.86	101.45	28.74	66.25	45.15	94.18	23.59	81.79	47.10
Cl^–^	147.85	92.20	136.55	119.95	183.84	144.30	160.58	152.32	182.53	157.25	125.25	123.67
K^+^	35.81	22.65	35.22	30.83	42.41	24.12	38.98	33.52	39.42	20.79	41.03	34.35
PO_4_^3^^–^	17.81	0.00	23.65	0.00	52.79	0.00	22.52	0.00	40.18	0.00	25.49	0.00

*Standard deviation was less than 5% in all operative conditions. **Where-IC**, initial concentration; **FC**, final concentration.*

**FIGURE 5:**
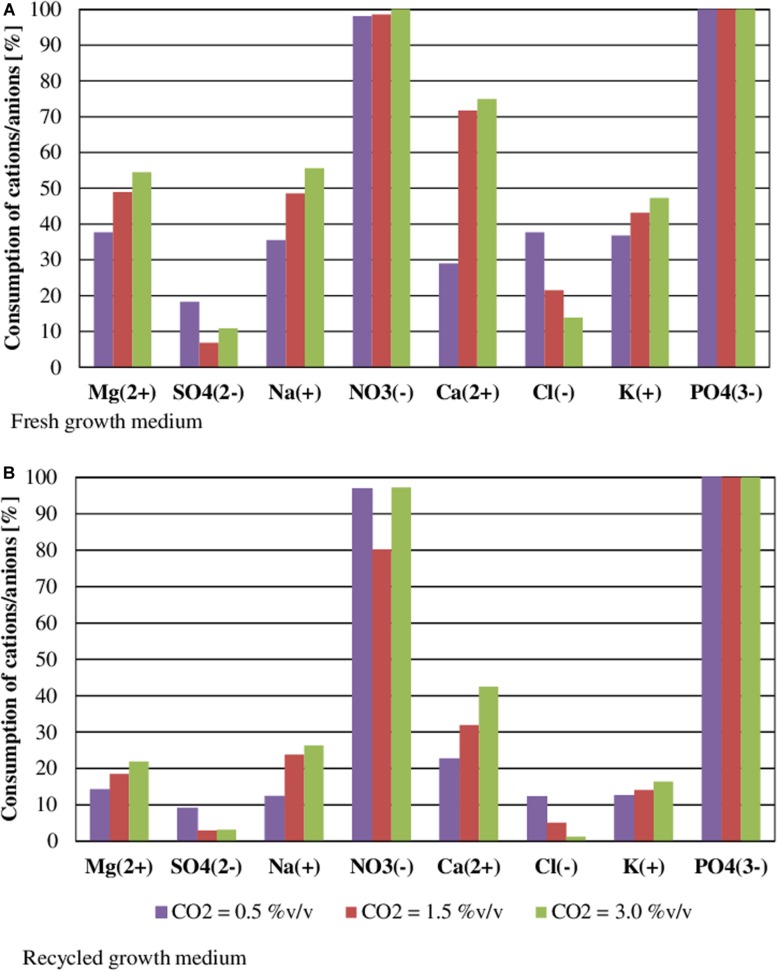
Effect of CO_2_ content on nutrient consumption during the cultivation of *S. almeriensis* with fresh growth medium **(A)** and with recycled growth medium **(B)**. Standard deviation was calculated on five technical replications and it was less than 5% in all operative conditions.

Figure 5 shows nutrient consumption during the cultivation of *S. almeriensis* with fresh and reused growth medium in different CO_2_ contents. N and P were highly consumed in all experimental conditions, as they are the main contributors to sustain microalgae growth (Lam et al., 2012; Choi et al., 2017). At the end of the experiments PO_4_^3^^–^ was totally used up in every CO_2_ concentration with both fresh and reused growth media, even if its initial concentration was varied in the range 17.81–52.79 mg/L (Table 3). This may suggest that PO_4_^3^^–^ is necessary for *S. almeriensis* growth, which confirms the results by Hadj-Romdhane et al. (2012). A similar consideration can be put forward for NO_3_^–^: 98–100% consumption rate with the fresh medium and a slightly lower rate (80–97%) with the recycled medium.

Results in Table 3 indicate that the higher the CO_2_ content the higher the consumption of nutrients, except for Cl^–^ and SO_4_^2^^–^. The consumption of Cl^–^ and of SO_4_^2^^–^ was lower than 37 and 18%, respectively, with both the fresh and the recycled growth medium for all the investigated CO_2_ contents, this results suggests that by growing *S. almeriensis* in the presence of CO_2_ the biomass produced has a different composition and in particular a lower amount of Cl^–^ and SO_4_^2^^–^. Similar results were obtained in tests of *Chlorella vulgaris* cultivation by growth medium recycling (Hadj-Romdhane et al., 2013). The consumption of Ca_2_^+^, Cl^–^, K^+^, Mg_2_^+^, Na^+^, NO_3_^–^, and SO_4_^2^^–^ was reduced up to 40% using the recycled medium. Remarkably, during the growth with a CO_2_ content of 0.5%v/v, the consumption of Cl^–^ was around 37 and 12% with the fresh and the reused growth medium, respectively. During the growth with the recycled medium, an increase in osmolality can be found, leading to a decrease of the consumption of nutrients (Hadj-Romdhane et al., 2012). Therefore, the recycled growth medium could limit the growth of *S. almeriensis*, by decreasing the microalgae nutrient utilization.

Results of statistical analysis are reported in Table 4. Significant differences in nutrient consumption were observed at all CO_2_ contents and for all nutrients, except NO_3_^–^, between fresh and recycled growth media. No statistically significant differences were observed in NO_3_^–^ consumption at 0.5 and 3% v/v CO_2_ contents.

**TABLE 4:** Nutrient consumption at different CO_2_ contents with fresh and with recycled growth medium: ANOVA (one-way; α = 0.05) results.

	***p-value***		
	**CO_2_ concentration (%v/v)**		
**Nutrients**	**0.5**	**1.5**	**3**
Mg^2 +^	0.000022	0.000022	0.000026
SO_4^2–_	0.000065	0.000035	0.000012
Na^+^	0.000019	0.000047	0.000041
NO_3–_	0.90	0.002564	0.37
Ca^2 +^	0.002159	0.000033	0.000099
Cl^–^	0.000014	0.000008	0.000004
K^+^	0.000016	0.000015	0.000018

### CO_2_ Content and Recycled Medium Effects on Extraction Yield and Lutein Production

**FIGURE 6:**
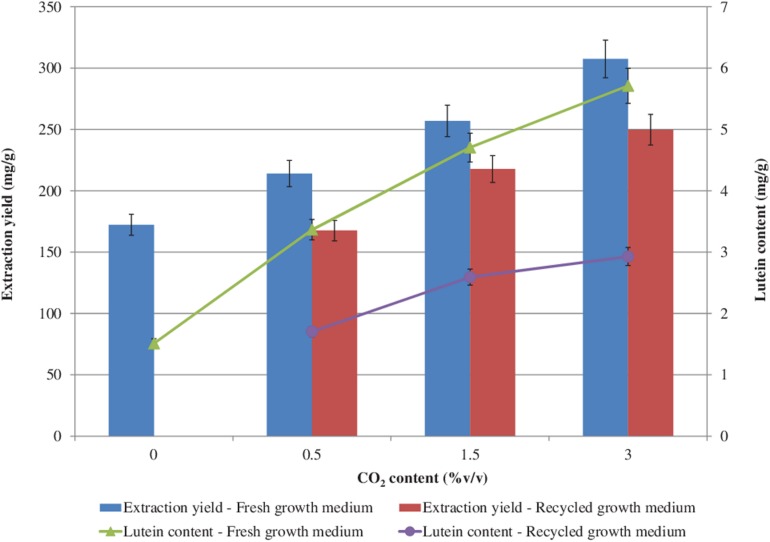
Extraction yield and lutein content (based on dry biomass weight) from *S. almeriensis* at different CO_2_ contents with fresh growth medium and with recycled growth medium. Standard deviation was calculated on five technical replications.

The effects of CO_2_ and of the recycled growth medium on the extraction yield and lutein content are shown in Figure 6. The higher the CO_2_ concentration the higher the extraction yield and the lutein content, both with the fresh and with the recycled growth medium. However, with the recycled growth medium, slightly lower extraction yield and lutein content values were found with respect to the growth with the fresh one. The highest extraction yield (307.44 mg/g) and the highest lutein content (5.71 mg/g) were achieved with a CO_2_ content of 3.0%v/v with the fresh growth medium. With the recycled growth medium, a 1.3-fold decrease in the extraction yield and a 2-fold decrease in the lutein content (maximum reductions) were measured, which might be attributed to the lower photosynthetic activity observed during the cultivation with the recovered growth medium (Hadj-Romdhane et al., 2012, 2013).

A slightly lower lutein content (5.56 mg/g) was obtained with 150 mg/L ammonium-N from *Desmodesmus* sp. F51 (Xie et al., 2017). Chen and Liu (2018) evaluated the effect of medium replacement during a two-stage cultivation process of *Chlorella sorokiniana* MB-1. The 60% replacement of the effluent from the first stage to the second stage with the fresh medium resulted in a lutein content below 3.5 mg/g, while the 80% replacement of the effluent showed a lutein concentration lower than 2.5 mg/g (Chen and Liu, 2018).

Results of statistical analysis are reported in Table 5. Significant differences in extraction yield and lutein extraction were observed between fresh and recycled growth media at all CO_2_ contents.

**TABLE 5:** Extraction yield and lutein extraction at different CO_2_ contents with fresh and with recycled growth medium: ANOVA (one-way; α = 0.05) results.

	***p-value***	
**CO_2_ concentration (%v/v)**	**Extraction yield**	**Lutein extraction**
0.5	0.0042	0.00010
1.5	0.0151	0.00017
3	0.0073	0.00013

The lutein content obtained in this study, equal to 5.71 mg/g, was lower than the value of 8.54 mg/g reported by Molino et al. (2019) who investigated the performance of the same microalgal species using a vertical bubble column photo-bioreactor with a volume to surface ratio (V/S) of 56.6 L/m^2^. The different lutein content can be explained by the different photo-bioreactor configuration in the two research works: V/S ratio = 11.5 L/m^2^ (this work) and = 56.6 L/m^2^ (Molino et al., 2019). On the other hand, the value reported in this work is slightly higher than those reported in microalgae-based lutein studies using batch phototrophic conditions (Sánchez et al., 2008b; Ho et al., 2014; Xie et al., 2014, 2017), while it is comparable with the values reported by Sánchez et al. (2008a, b) who studied *S. almeriensis* growth in a continuous mode. Ho et al. (2014) optimized the effect of light intensity founding that 75 μmol/m^2^/s was the optimum choice to attain higher lutein contents with continuous aeration of 2.5%v/v CO_2_; the maximum lutein content of 5.52 mg/g from *Scenedesmus obliquus* FSP-3 was produced by supplying 8.0 mM calcium nitrate as the nitrogen source during cultivation (Ho et al., 2014). Dineshkumar et al. (2015a) cultivated *Chlorella minutissima* MCC-27 in a 2-L airlift photo-bioreactor and achieved a maximum lutein productivity of 3.45 mg/L/d in batch system. Moreover, lutein productivity of *Chlorella minutissima* MCC-27 could be further enhanced to 4.32 mg/L/d by optimization of the operative parameters such as light intensity, CO_2_ concentration and gaseous flow rate (Dineshkumar et al., 2015a). These observations highlight the importance of the photo-bioreactor configuration choice for the selected microalgae strain.

A comparison between the lutein content extracted in this study and the literature results is reported in the following table (Table 6):

**TABLE 6:** Lutein content: microalgae comparison (adapted from Molino et al., 2019).

**Microalgal**	**Lutein content**	**References**
**strain**	**(mg/g dry biomass)**	
*Chlorella sorokiniana*	3.0	Cordero et al., 2011
*Scenedesmus obliquus* FSP-3	4.52	Ho et al., 2014
*Chlorella zofingiensis*	7.2	Del Campo et al., 2004
*Scenedesmus almeriensis*	5.5	Sánchez et al., 2008a
*Scenedesmus almeriensis*	5.3	Sánchez et al., 2008b
*Desmodesmus* sp. F51	5.5	Xie et al., 2013
*Coccomyxa onubensis*	6.2	Vaquero et al., 2012
*Muriellopsis* sp.	4.3	Del Campo et al., 2007
*Chlorella zofingiensis*	3.4	Del Campo et al., 2007
*Chlorella minutissima*	6.37	Dineshkumar et al., 2015a
*Chlorella minutissima* MCC-27	6.05	Dineshkumar et al., 2015b
*Scenedesmus almeriensis*	8.54	Molino et al., 2019
*Chlorella sorokiniana* MB-1	3.5 (60% recycled medium)	Chen and Liu, 2018
	2.5 (80% recycled medium)	
*Scenedesmus almeriensis*	5.71 (fresh medium)	This study
	2.93 (recycled medium)	

## Conclusion

The effects of the fresh and the recycled growth medium, at different CO_2_ concentrations on the productivity and the concentration of *S. almeriensis*, and on the lutein production were investigated. The recycled medium was found to limit the growth of *S. almeriensis*, as a decreased amount of available nutrients was observed, which corresponded to a slight decrease in the biomass productivity and the lutein content. On the other hand, *S. almeriensis* resulted to be potentially effective for CO_2_ bio-fixation with promising performance rates; in particular, the higher the CO_2_ content the higher the biomass productivity, as well as the microalgae concentration and the lutein content. *S. almeriensis* could be an ideal candidate for the commercial production of lutein from microalgae and for bio-fixation of CO_2_ at the same time.

## Data Availability Statement

All datasets generated for this study are included in the article/Supplementary Material.

## Author Contributions

DM and AM: conceptualization. SM, AI, and DM: data curation. AI: formal analysis. SM: investigation. AI, PC, DK, and SC: methodology. SM and TM: writing – original draft. SM: writing – review draft. AM: project administration and resources. DM and AM: supervision.

## Conflict of Interest

The authors declare that the research was conducted in the absence of any commercial or financial relationships that could be construed as a potential conflict of interest.
