# Supplementary figures and images for: Enhancing Biomass and Lutein Production From Scenedesmus almeriensis: Effect of Carbon Dioxide Concentration and Culture Medium Reuse
Source: Front Plant Sci. 2020 Apr 21;11:415. doi: 10.3389/fpls.2020.00415 (PMC7186383; doi:10.3389/fpls.2020.00415)

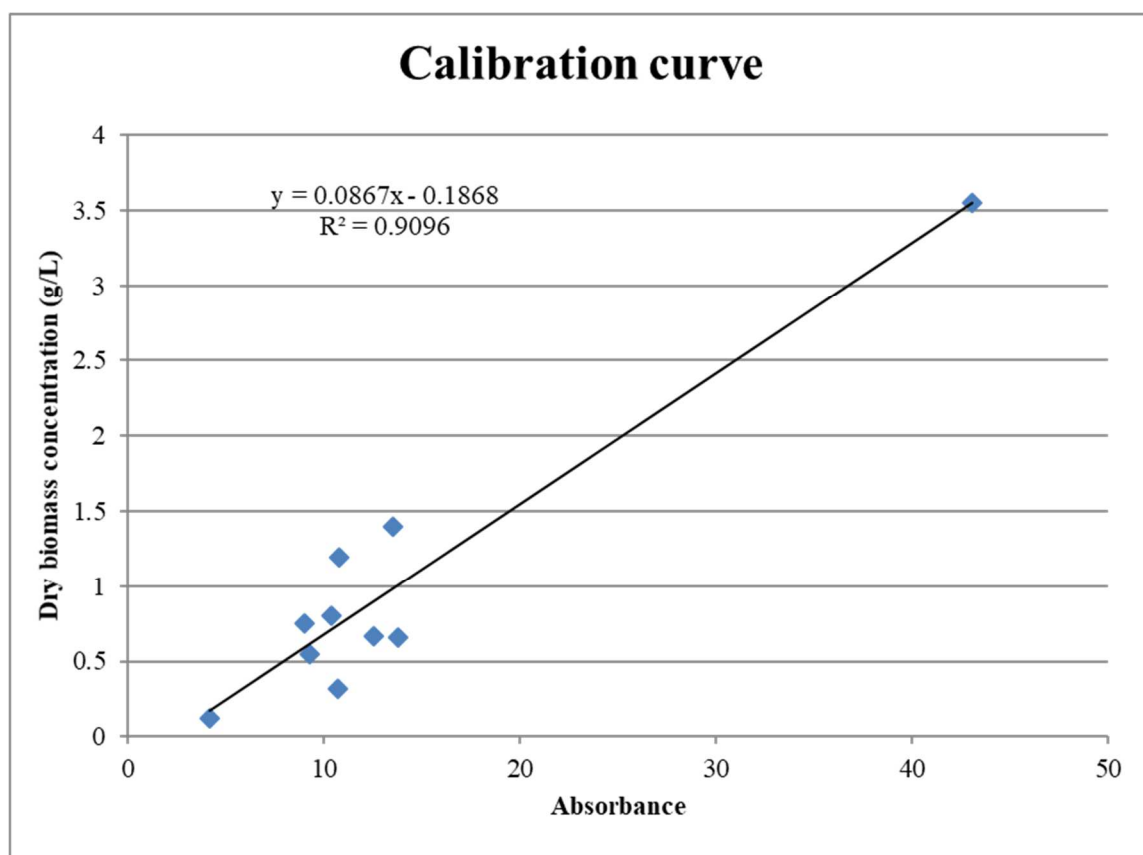

Supplementary Figure 1. Calibration curve

Supplement: Supplementary file 1 [file Image_1.pdf]
